# Case Report: Intracranial peripheral primitive neuroectodermal tumor – Ewing's sarcoma of dura with transcalvarial–subgaleal extension: An unusual radiological presentation

**DOI:** 10.4103/0971-3026.57215

**Published:** 2009-11

**Authors:** Shahina Bano, Sachchida Nand Yadav, Umesh Chandra Garga

**Affiliations:** Department of Radiodiagnosis, Dr Ram Manohar Lohia Hospital and PGIMER, New Delhi - 110 001, India

**Keywords:** Computed tomography (CT), intracranial pPNET-ES of dura, magnetic resonance imaging (MRI)

## Abstract

The occurrence of the intracranial, peripheral, primitive, neuroectodermal tumor, Ewing's sarcoma (pPNET-ES) of the dura, is very rare. Immunophenotypical as well as genetic analyses play key roles in its diagnosis and differentiation from central PNET. We describe here the CT scan and MRI findings of an interesting case of intracranial pPNET-ES arising from the anterior falx cerebri with a trancalvarial–subgaleal extension.

## Introduction

The peripheral, primitive neuroectodermal tumor (pPNET), Ewing's sarcoma (ES) family tumor group, includes small round cell tumors of the bone, nerve, and soft tissues with morphological attributes of the germinal neuroepithelium. pPNET-ES also occurs in the central nervous system (CNS), including the meninges and the cranial and spinal nerve roots.[1] The distinction between pPNET-ES of the CNS and a central primitive neuroectodermal tumor (cPNET), including the infratentorial medulloblastoma, supratentorial cerebral neuroblastoma, and pineoblastoma, is important because the two entities require distinct treatments and carry different prognoses. However, this distinction is not possible on imaging and requires immunohistochemistry and molecular genetic analysis. Because of the small number of patients, the prognosis of pPNET-ES is not clearly known although it has been suggested that patients with pPNET-ES arising from the structures within or around the CNS may have more favorable outcomes than patients with c-PNET.[2]

## Case Report

**Figure 1 (A-G):**
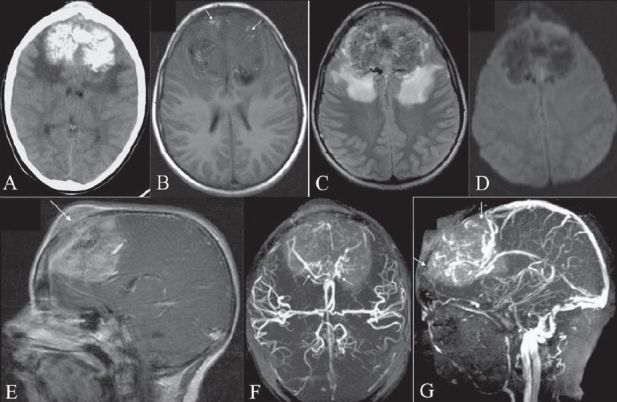
Plain CT scan (A) demonstrates an extensively calcified, hyperdense, parafalcine, extra-axial mass. Axial T1W MRI image (B) reveals the mass to be predominantly hypointense with hyperintense calcifications (arrows). An axial T2W MRI image (C) shows it to be heterogeneously isohyperintense with marked perifocal edema. An axial diffusion-weighted MRI image (D) reveals lack of restriction of diffusion within the tumor. A contrast-enhanced T1W, sagittal MRI image (E) reveals diffuse heterogeneous enhancement with transcalvarial–subgaleal extension (arrows). A three-dimensional time-of-flight MRI angiography image (F) reveals extensive tumor neovascularity with splaying and posterolateral displacement of both anterior cerebral arteries (arrows). An MRI venography image (G) demonstrates thrombosis/invasion of the anterior one-third of the superior sagittal sinus (arrow)

An 11-year-old girl presented with a 3-month history of a midline, frontal, non-tender scalp swelling with a rapid and recent increase in size. She had repeated episodes of headache, giddiness, and mild behavioral changes. In addition, epiphora, diplopia, and blurring were also present. Fundoscopy revealed bilateral papilledema. No focal neurological deficit was detected. Non-enhanced CT scan of the brain revealed a large 7.0 × 5.0 × 5.0 cm, densely calcified, mushroom-shaped, parasagittal, hyperdense mass arising from the anterior falx [Figure 1A]. The non-calcified portion of the tumor showed marked enhancement following intravenous administration of contrast. There was destruction of both the inner and the outer tables of the overlying calvarium with transcalvarial–subgaleal extension. Extensive perilesional, vasogenic, white matter edema was also noted. On MRI, the lesion appeared predominantly hypointense on T1W images with internal hyperintense areas corresponding to calcifications on CT scans [Figure 1B] and heterogeneously isohyperintense on T2W images [Figure 1C]. Diffusion-weighted images and corresponding ADC mapping did not demonstrate any area of restricted diffusion [Figure 1D]. Heterogeneous enhancement was seen on the post-gadolinium T1W images [Figure 1E]. MRI angiography revealed extensive tumor neovascularity with splaying and posterolateral displacement of the anterior cerebral arteries, bilaterally, by the tumor [Figure 1F]. Consistent with thrombosis/invasion, the anterior one-third of the superior sagittal sinus (SSS) was not visualized on MRI venography [Figure 1G]. MRI spectroscopy was not informative due to excessive calcification and high vascularity. MRI of the entire spine was negative for tumor metastases and a thoraco-abdominal CT scan was also unremarkable.

The patient underwent subtotal resection of the tumor. Intra-operative findings confirmed the dural origin, extensive calcifications, high vascularity, and aggressive nature of the tumor with invasion of the adjoining brain parenchyma along all the margins. There was destruction of the overlying calvarium with an associated subgaleal mass. The anterior one-third of the SSS was also found to be thrombosed and encased by the tumor.

**Figure 2 (A, B):**
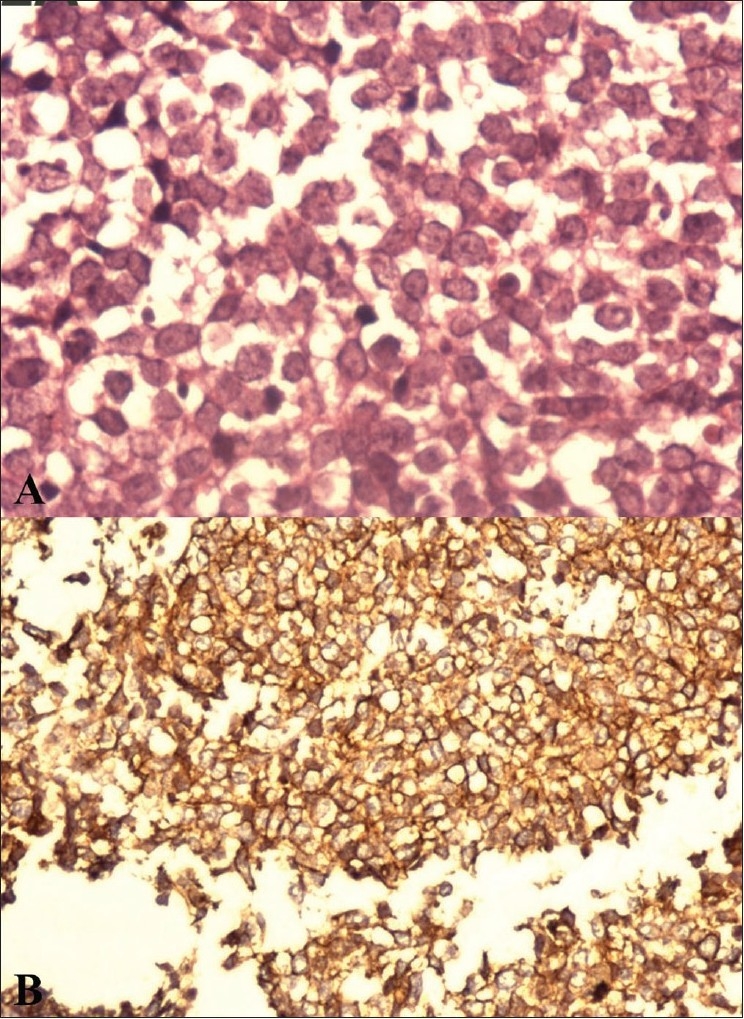
Histopathology image (A) shows a cluster of uniform, small round cells with fine, pale chromatin and moderate amount of cytoplasm with no intercellular matrix (hematoxylin and eosin-stained section). CD99 immunohistochemistry image (B) shows strong membranous staining (original magnification, X400)

Histopathological examination revealed a cluster of undifferentiated round cells [Figure 2A]. On immunohistochemical and fluorescent *in situ* hybridization analyses, the tumor showed CD99 [Figure 2B], *MIC-2* antigen expression, and chromosomal translocation t(11:22)(q24:q12).

## Discussion

pPNET-ES primarily affects children and young adults aged 10–30 years with equal sex predilection.[3] Although pPNET-ES has a predilection for bone and soft tissue, it can arise virtually in any location. Involvement of the intracranial compartment is rare, but if this lesion arises intracranially, it is commonly misdiagnosed as c-PNET because of the similarity in the histological appearance. Histologically, the tumor exhibits primitive, undifferentiated round cell morphology.

Recent advances in molecular biology have allowed a clear pathological distinction between these two entities. The *MIC2* gene product (CD99) is highly expressed immunohistochemically in nearly all pPNET-ES.[4] Central PNETs are reported to be negative for CD99 staining.[5] The chromosomal translocation t(11, 22)(q29; q12) is found in >90% of pPNET-ES and appears to be characteristic,[6] but is not found in primary cerebral and cerebellar PNET.[7] Our case also demonstrated both CD99 staining and the characteristic chromosomal translocation.

There is sparse literature regarding the radiological features of pathologically proven intracranial pPNETs. In 2006, Pekala *et al*,[8] showed restriction of diffusion in both of their cases, unlike in our patient. In 2001, Dick *et al*,[9] reported that non-CNS pPNETs tend to displace adjacent soft tissue structures rather than invade or encase them and generally have sparse calcification. It is possible, as in our patient, that CNS pPNETs behave differently.

It is not possible to make a prospective, definitive diagnosis of this condition on imaging. A lesion such as seen in our patient would have a differential diagnosis that would include c-PNET, aggressive meningioma, rhabdomyosarcoma, and esthesioneuroblastoma.

In summary, we describe here a rare case of intracranial pPNET-ES of the dura with transcalvarial–subgaleal extension. Although it is only possible to make this diagnosis on immunohistochemistry and genetic analyses, knowledge of this condition will help radiologists understand the spectrum of intracranial round cell neoplasms better.
